#  Integrated bioinformatics analysis revealed the regulation of angiogenesis by tumor cells in hepatocellular carcinoma

**DOI:** 10.1042/BSR20210126

**Published:** 2021-07-01

**Authors:** Meisi Huo, Kangkang Yu, Yahui Zheng, Lu Liu, Hao Zhao, Xiaoqi Li, Chong Huang, Jubo Zhang

**Affiliations:** Department of Infectious Diseases, Shanghai Key Laboratory of Infectious Diseases and Biosafety Emergency Response, Huashan Hospital, Fudan University, Shanghai 200040, China

**Keywords:** angiogenesis, bioinformatic analysis, GEO, hepatocellular carcinoma, microRNA

## Abstract

Hepatocellular carcinoma (HCC) is one of the leading causes of cancer mortality, metastasis accounts for most of the cases. Angiogenesis plays an important role in cancer metastasis, but how tumor cells affect the function of endothelial cells by dictating their microRNA (miRNA) expression remains largely unknown.

Differentially expressed miRNAs (DEMs) were identified through dataset downloaded from the Gene Expression Omnibus (GEO) database and analyzed by GEO2R. We then used online tools to obtain potential targets of candidate miRNAs and functional enrichment analysis, as well as the protein-protein interaction (PPI). Finally, the function of miR-302c-3p was validated through *in vitro* assay.

In the current study, we found that HCC cells altered miRNA expression profiles of human umbilical vein endothelial cells (HUVECs) and miR-302c-3p was the most down-regulated miRNA in HUVECs when they were co-cultured with HCC-LM3 cells. Functional enrichment analysis of the candidate targets revealed that these genes were involved in epigenetic regulation of gene expression, in particular, cytosine methylation. In addition, PPI network demonstrated distinct roles of genes targeted by miR-302c-3p. Importantly, inhibition of angiogenesis, migration and permeability by the most down-regulated miR-302c-3p in HUVECs was confirmed *in vitro*. These findings brought us novel insight into the regulation of angiogenesis by HCC cells and provided potential targets for the development of therapeutic strategies.

## Introduction

Hepatocellular carcinoma (HCC) is the sixth most common occurring cancer and the fourth leading cause of cancer mortality worldwide [1]. Most cancer-related deaths result from metastasis, which accounts for about 90% deaths [2,3]. Actually, the high incidence of metastasis and recurrence usually resulted in poor clinical prognosis of HCC patients [4].

Angiogenesis is the growth of blood vessels from the existing vasculature [5]. In adults, blood vessels remain quiescent and rarely form new branches under physiological conditions. However, tumor develop a vascular network rapidly by angiogenesis to support the high proliferative rate of cancer cells [6]. Angiogenesis has been implicated in cancer metastasis and recurrence for a long time [7–10], but how tumor cells regulate endothelial cells to orchestrate angiogenesis remains poorly understood.

MicroRNAs (miRNAs) are a class of small non-coding RNAs of ∼20 nucleotides in length, and generally repress gene expression by binding to the 3′ UTR of target gene’s mRNA [11]. MiRNAs have been recognized as critical regulators of physiological and pathological processes, including carcinogenesis and tumor progression [12,13]. In HCC, it has been reported that miR-193a-5p inhibited hepatocarcinogenesis by targeting NUSAP1 [14]. miR-122 could promote HCC cell apoptosis through directly silencing the biogenesis of miR-21 [15]. miR-199a-3p suppressed HCC cell proliferation and migration by down-regulating mTOR [16]. In HCV-associated hepatocarcinogenesis, miR-135a-5p promoted tumor progression by inhibiting PTPRD expression [17]. In TGF-β-mediated liver cancer cell migration, miR-449 family played inhibitory roles by targeting SOX4 [18].

However, studies on the regulation of endothelial cell function by miRNAs dictated by hepatoma cells are limited. Zhu and colleagues previously focused on miRNAs up-regulated by hepatoma cells found that miR-146a promoted angiogenesis in HCC by increasing PDGFRA expression [19]. However, the function of these miRNAs down-regulated by HCC cells is of great importance as well, and thus should not be ignored.

Here, by analyzing public dataset GSE44567, we explored miRNAs expression alteration in human umbilical vein endothelial cells (HUVECs) under HCC-LM3 exposure and identified down-regulated miR-302c-3p as the only miRNA showed significant differential expression. Targets of miR-302c-3p were predicted by four online tools, and common genes predicted by these web tools were selected as candidate targets. To get insight into the function of the candidate targets, functional enrichment analysis was performed. In addition, protein-protein interaction (PPI) network of the candidate targets was established followed by identification of hub genes, whose expressions were evaluated using single cell RNA-seq data. Finally, the inhibitory role of miR-302c-3p on angiogenesis, migration and permeability was validated *in vitro*.

## Methods

### Microarray dataset

The GSE44567 dataset, which based on the platform of GPL11487 (Agilent-021827 Human miRNA Microarray), was downloaded from the Gene Expression Omnibus database (GEO, https://www.ncbi.nlm.nih.gov/geo/). The dataset contains miRNAs expression profiles of HUVECs exposed to HCC-LM3 cells (*n*=4) or not (*n*=4). To identify differentially expressed miRNAs (DEMs), GEO2R (https://www.ncbi.nlm.nih.gov/geo/geo2r/) was engaged and adjusted *P*-value <0.05 was set as the threshold to identify DEMs.

### Target prediction and functional enrichment analysis

Four online tools: miRDB [20] (http://mirdb.org/), TargetScan [21] (http://www.targetscan.org/vert_72/), mirDIP (http://ophid.utoronto.ca/mirDIP/) and microT-CDS [22] (http://diana.imis.athena-innovation.gr/DianaTools/index.php?r=microT_CDS/index), were engaged to predict potential targets of miR-302c-3p. Shared genes obtained from the four tools were selected as candidate targets and were subjected to Metascape for functional enrichment analysis.

### PPI network establishment and hub gene identification

STRING database (https://string-db.org/) was used to construct the PPI of the potential targets of miR-302c-3p. The interaction network was visualized by Cytoscape (Version 3.7.1), and genes with strongest connectivity were selected as hub genes. Expressions of hub genes were evaluated by querying human liver cell atlas (http://human-liver-cell-atlas.ie-freiburg.mpg.de/).

### Cell culture and transfection

HUVECs were kept in DMEM with 10% FBS. The miR-302c-3p mimics and inhibitors were purchased from RiboBio. Transfection was performed using Lipofectamine 2000 and Opti-MEM as per the manufacturers’ instruction.

### Expression of target genes

Western blot and RT-qPCR were performed as previously described [23].

The following primary antibodies were used: APP, amyloid-β precursor protein (Proteintech, 25524-1-AP, 1:500 dilution), GAPDH (Proteintech, 60004-1-Ig, 1:20000 dilution).

The primer sequences were as follows: GAPDH, F: 5′-AGAAGGCTGGGGCTCATTTG-3′; R: 5′-AGGGGCCATCCACAGTCTTC-3′; APP, F: 5′-TCTCGTTCCTGACAAGTGCAA-3′; R: 5′-GCAAGTTGGTACTCTTCTCACTG-3′.

### Tube formation assay

Treated HUVECs were seeded into 24-well plate pretreated with Matrigel matrix, and then incubated at 37°C in 5% CO_2_ humidified atmosphere. Tube formation was observed at 12h with microscope. The tube formation ability was evaluated by counting the number of tubes. Each experiment was repeated three times. Tube formation was quantified with Image J.

### Transwell migration assay

Treated HUVECs (10^5^ per well) suspended in serum-free DMEM were added to the top well of the transwell chamber (8-μm pore size; Corning) and DMEM with 10% FBS were added into bottom of the well. After 12h incubation, the cells that penetrated through the membrane were fixed with 4% polyformaldehyde for 15 min and stained with 0.1% Crystal Violet solution, and then observed with microscope. The migration ability was evaluated by counting the number of penetrated cells. Each experiment was repeated three times.

### *In vitro* permeability assay

RITC-dextran (average MW ∼70000; Sigma) was added to the top well of the transwell chamber (0.4-μm pore size; Corning) on which treated HUVECs (10^5^ cells per well) were seeded for 72 h. After 30 min, the medium in the bottom well was collected and the appearance of fluorescence was monitored at 544 nm excitation and 590 nm emission [24]. Each experiment was repeated three times.

### Statistical analysis

Most statistical analyses were generated by online tools mentioned above. For tube formation quantification, Student’s *t* test was performed with GraphPad Prism 7. Data were presented as means ± SD, two-sided *P*-value <0.05 was considered significant.

## Results

### Identification of DEMs

**Figure 1:**
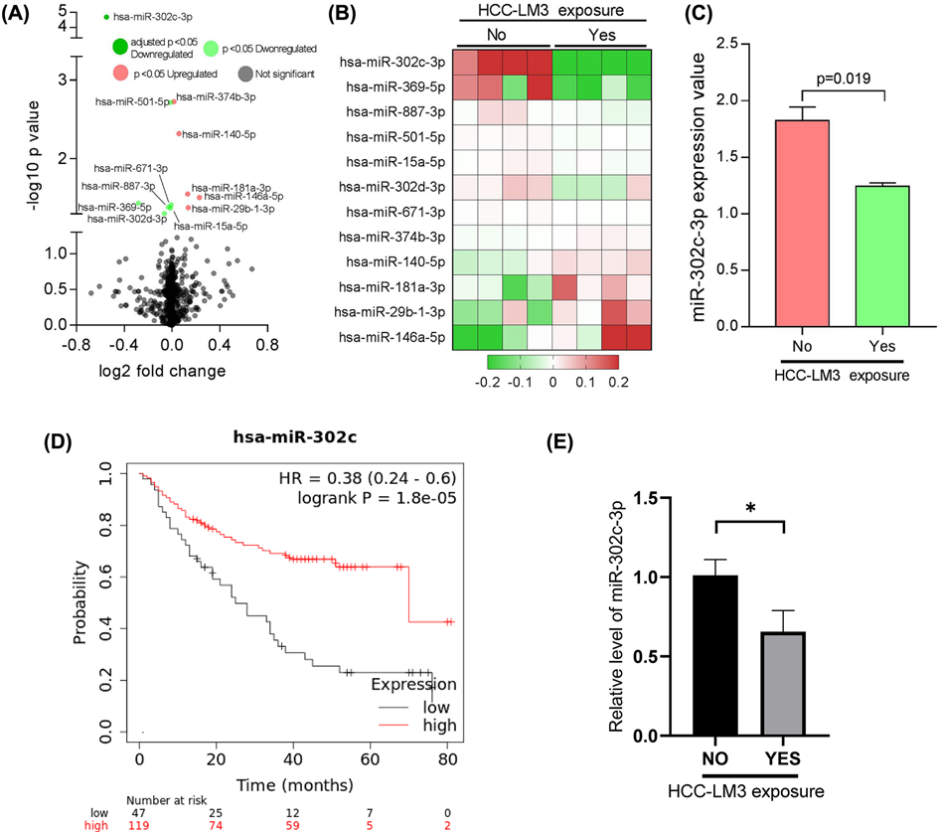
Identification of DEMs (**A**) Volcano plot shows the global miRNA expression alterations in HUVECs exposed to HCC-LM3. Up-regulated miRNAs were marked in red, while down-regulated miRNAs were marked in green. (**B**) Heatmap shows the expressions of the up- and down-regulated miRNAs. (**C**) Column graph shows miR-302c-3p expression value between HUVECs with and without HCC-LM3 exposure based on database. (**D**) Overall survival analysis using Kaplan-Meier Plotter with miR-302c-3p as an index. (**E**) Relative level of miR-302c-3p expression between HUVECs with and without HCC-LM3 exposure. *: *P*<0.05.

To identify DEMs, GSE44567 dataset was analyzed by GEO2R. Although 12 miRNAs were identified with *P*-value <0.05 (Figure 1A,B), only miR-302c-3p showed adjusted *P*-value <0.05 with log2 fold change of −0.549 (Supplementary Table S1). Comparison of miR-302c-3p expression between HUVECs with HCC-LM3 cells exposure and those without exposure was presented in Figure 1C. Results from Kaplan-Meier Plotter (http://www.kmplot.com/analysis) showed that liver cancer patients with high level of miR-302c-3p perform better survival outcome (Figure 1D). We also validated the miR-302c-3p expression with HCC-LM3 exposure, which was lower than control group (Figure 1E).

### Target prediction and functional enrichment analysis

**Figure 2:**
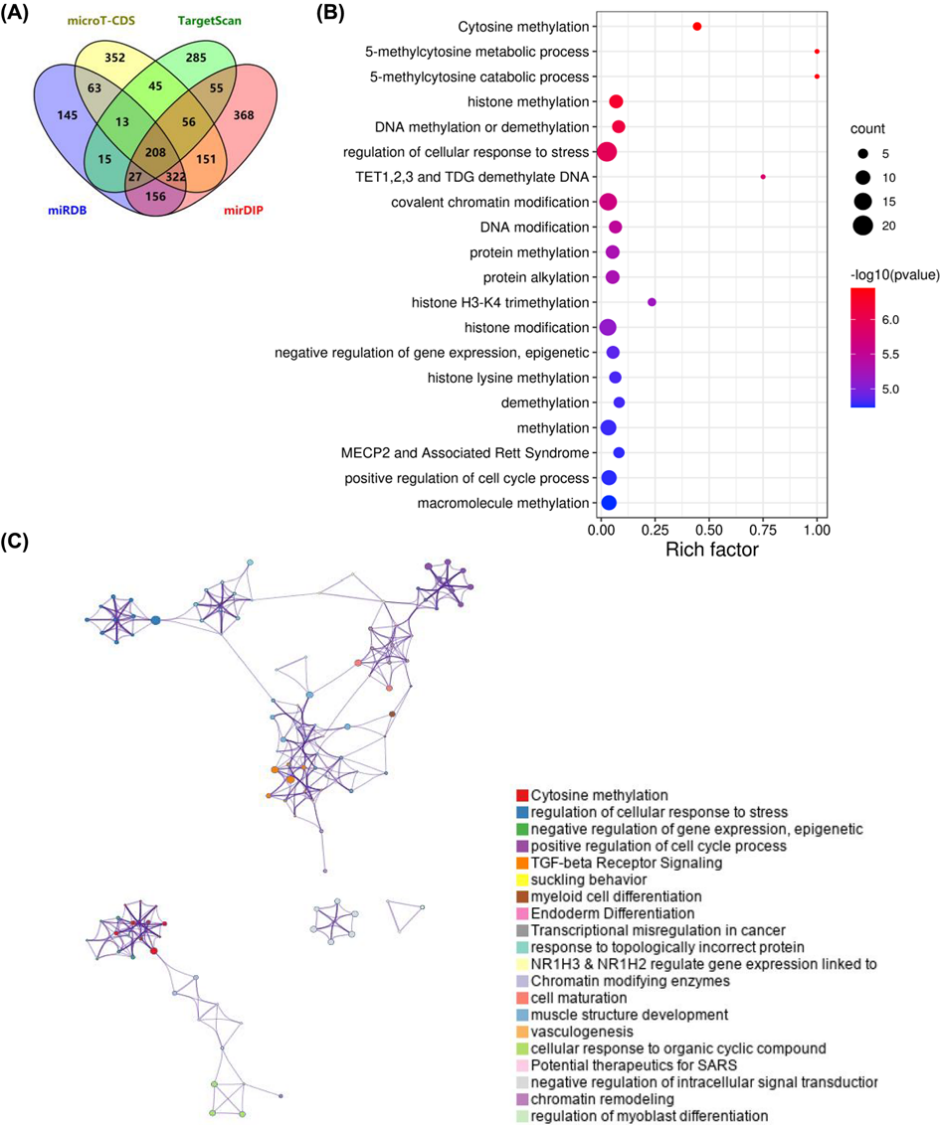
Targets prediction and functional enrichment analysis (**A**) Venn diagram shows the identification of 208 candidate target genes by intersecting four target genes from different online tools. Top 20 enriched terms (**B**) and grouped clusters (**C**) were obtained from functional enrichment analysis by subjecting candidate target genes to Metascape.

To obtain potential targets of miR-302c-3p, online tool miRDB, TargetScan, mirDIP and microT-CDS were adopted. The results showed that miRDB predicted 949 target genes, TargetScan predicted 704 target genes, mirDIP predicted 1343 target genes and microT-CDS predicted 1212 target genes. By intersecting the four target gene lists, 208 common genes were selected as candidate targets of miR-302c-3p (Figure 2A), and were then subjected to Metascape for functional enrichment analysis. The top 20 enriched terms were presented in Figure 2B, which indicated that histone and non-histone protein methylation were the most enriched terms. In addition, the enriched terms were grouped into clusters (Figure 2C and Supplementary Figure S1), and the top five clusters were: cytosine methylation, regulation of cellular response to stress, negative regulation of gene expression (epigenetic), positive regulation of cell cycle process and TGF-β receptor signaling.

### PPI analysis and hub genes identification

**Figure 3:**
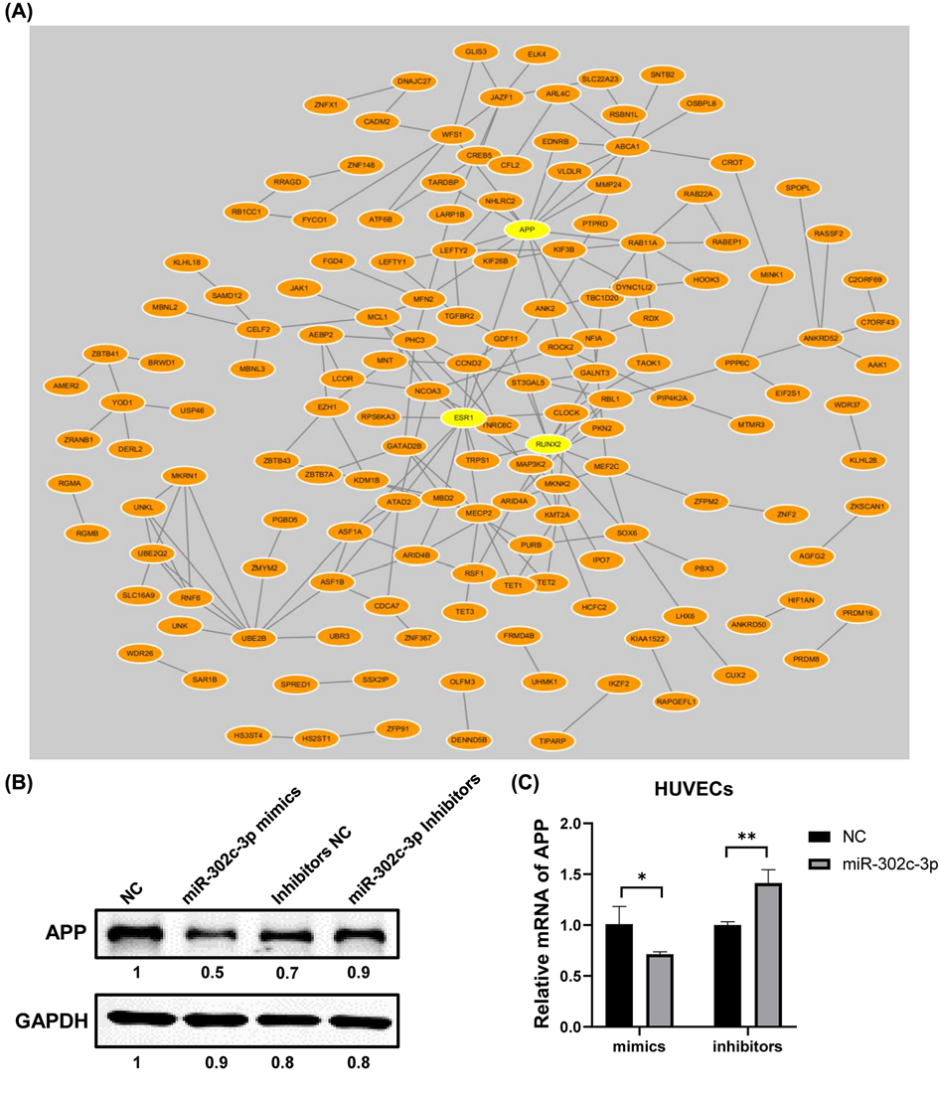
PPI analysis and hub genes identification (**A**) PPI network of the candidate target genes was visualized by Cytoscape and four hub genes with high node score and strong connectivity were marked in red. (**B,C**) Western blotting analysis and RT-qPCR analysis of APP in HUVECs after transfection. GAPDH was used as a loading control. *: *P*<0.05, **: *P* <0.01.

**Figure 4:**
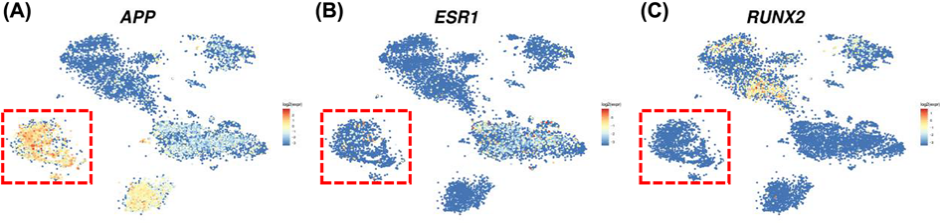
Expression of three hub genes based on single-cell RNA-seq data Expression of APP (**A**), ESR1 (**B**), RUNX2 (**C**), based on single-cell RNA-seq data. Clusters of liver sinusoidal endothelial cells, macrovascular endothelial cells and other endothelial cells were labeled within a red dotted box.

**Figure 5:**
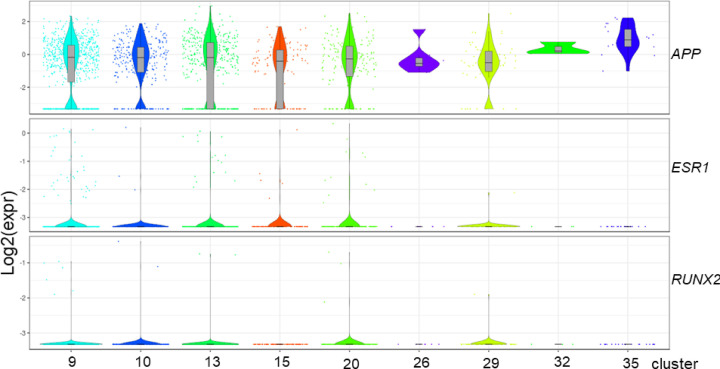
Expression of three hub genes in different clusters of endothelial cells based on single-cell RNA-seq data The violin graph shows expression of APP, ESR1, RUNX2 in endothelial cell clusters (cluster 9,10,13,15,20,26,29,32,35).

By querying STRING database, PPI network of the candidate target genes was established and then visualized by Cytoscape (Figure 3A). Three genes (*APP, ESR1, RUNX2*) with high node score and strong connectivity were selected as hub genes. We then evaluated the expression of the three hub genes based on single-cell RNA-seq data (Figure 4A-C), which revealed that only APP showed significant high expression in liver sinusoidal endothelial cells and macrovascular endothelial cells (Figures 4A and 5, Supplementary Figure S2). Then it was verified that APP could be modulated by miR-302c-3p on both protein and mRNA levels by Western blot and RT-qPCR assays (Figure 3B,C).

### MiR-302c-3p function validation

**Figure 6:**
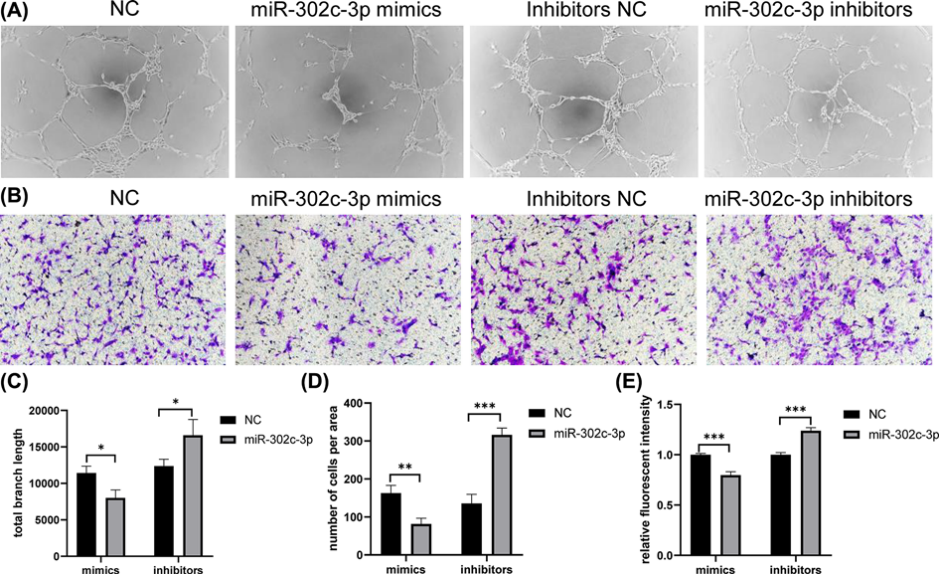
Function validation of miR-302c-3p HUVECs were transfected with miR-302c-3p mimics or inhibitors and the corresponding NC. (**A**) Effect of miR-302c-3p on angiogenesis was detected by tube formation assay. (**B**) Effect of miR-302c-3p on migration ability was detected by transwell migration assay. (**C**) Quantitative analysis of (A) (*n*=3). (**D**) Quantitative analysis of (B) (*n*=3). (**E**) Effect of miR-302c-3p on permeability of HUVEC monolayers by *in vitro* permeability assay (*n*=3). *: *P*<0.05, **: *P*<0.01, ***: *P*<0.001.

Given that miR-302c-3p was down-regulated in HUVECs by HCC-LM3, we suggested that miR-302c-3p was capable of inhibiting function of HUVECs. To confirm this hypothesis, we engaged tube formation assay, transwell migration assay and *in vitro* permeability assay. As expected, *in vitro* detection showed that miR-302c-3p mimics significantly suppressed tube formation, migration ability and permeability of HUVECs (Figure 6).

## Discussion

As one of the hallmarks of cancer, the processes of new vessel formation, namely, angiogenesis, is crucial to the progression of various cancers [6]. By secreting various pro-angiogenic factors, tumor cells create an abnormal vascular network, which results in poorly perfused tumors [6]. Endothelial cells play critical roles in angiogenesis by responding to these stimulus derived from tumor cells [25]. As post-transcriptional regulator of gene expression, miRNAs have been implicated in cancer progression, especially in angiogenesis involving tumor metastasis [13,26].

In the current study, we conducted differential expression analysis of miRNAs in response to HCC-LM3 exposure using microarray data obtained from GEO database. We identified miR-302c-3p as the only DEM that down-regulated in HUVECs when cocultured with HCC-LM3. Functional enrichment analysis of the candidate targets of miR-302c-3p revealed that these genes were mainly involved in epigenetic regulation of gene expression, including chromatin modification, histone methylation and histone modification. One of the candidate target TET1 is an important regulator of DNA methylation, and has been identified as a critical oncoprotein [27,28].

By constructing PPI network, APP, ESR1 and RUNX2 were identified as hub genes. We then evaluated the expression of the three hub genes based on single-cell RNA-seq data [29], which was performed based on ∼10000 cells from normal liver tissue from nine human donors. The research team identified previously unknown subtypes of endothelial cells, Kupffer cells and hepatocytes with transcriptome-wide zonation and also provided a powerful resource to help the discovery of previously unknown cell types in normal and diseased livers [29]. APP, the amyloid-β precursor protein, is central to the pathogenesis of Alzheimer’s disease [30]. It has been reported that accumulation of APP in mitochondria can impair mitochondrial function and lead to obesity [31], a potential driver of various cancers [32–35]. ESR1 is an effective therapeutic target in breast cancer [36]. Although the role in HCC has not been elucidated, ESR1 was implicated in regulation of VEGFA, a key regulator of angiogenesis [37]. RUNX2 played an important role in the regulation of cancer cell invasion and migration, and recent studies revealed the involvement of RUNX2 in microenvironment remodeling [38,39].

Actually, miR-302c-3p has been implicated in various cancers. In testicular germ cell tumors, miR-302c-3p may act as an oncogene [40]. While in human endometrial carcinoma, miR-302c-3p inhibited epithelial-mesenchymal transition and promoted apoptosis of cancer cells [41]. In brain tumors, miR-302c-3p can inhibit invasion and proliferation of glioma cells by targeting MTDH [42]. In colorectal cancers (CRCs), miR-302c-3p inhibited CRC cell growth [43]. In HCC, previous studies revealed that miR-302c-3p repressed migration and invasion of HCC cells by targeting TRAF4 [44]. Here, we confirmed that miR-302c-3p, an miRNA down-regulated by hepatoma cells, can also inhibit angiogenesis, migration ability and *in vitro* permeability of HUVECs, the critical elements of tumor metastasis [45].

However, there are also some limitations in our study. First, we did not explain how miR-302c-3p in the endothelial cells were affected by HCC cell line and what intercellular communication happened between the cells. Second, the function of the miR-302c-3p was focused on *in vitro* assay only, which need to be further investigated *in vivo*.

In summary, by integrated bioinformatics analysis, we identified miR-302c-3p as the most down-regulated miRNA in HUVECs by HCC cells. Targets of miR-302c-3p were predicted and subsequent functional enrichment analysis provided us insight into the function of the candidate target genes. Hub genes obtained from PPI network displayed important role in regulating angiogenesis, tumor cell migration and invasion, and mitochondrial function. Further investigation of the function of miR-302c-3p and the hub genes will offer novel therapeutic targets for antiangiogenic therapy in HCC.

## Supplementary Material

Supplementary Figures S1-S2 and Table S1

## Data Availability

The dataset GSE44567 for the present study can be found in the GEO database (https://www.ncbi.nlm.nih.gov/geo/).

## Abbreviations

APP: amyloid-β precursor protein
DEM: differentially expressed miRNA
GAPDH: glyceraldehyde-3-phosphate dehydrogenase
GEO: Gene Expression Omnibus
HCC: hepatocellular carcinoma
HUVEC: human umbilical vein endothelial cell
miRNA: microRNA
PPI: protein-protein interacrion
RT-qPCR: quantitative reverse transcription PCR
