# Supplementary material for: Integrated bioinformatics analysis revealed the regulation of angiogenesis by tumor cells in hepatocellular carcinoma
Source: Biosci Rep. 2021 Jul 1;41(7):BSR20210126. doi: 10.1042/BSR20210126 (PMC8252189; doi:10.1042/BSR20210126)
Supplement: Supplementary Figures S1-S2 and Table S1 [file BSR-2021-0126_supp.pdf]

Figure S1: Grouped clusters of enriched terms based on functional enrichment analysis.

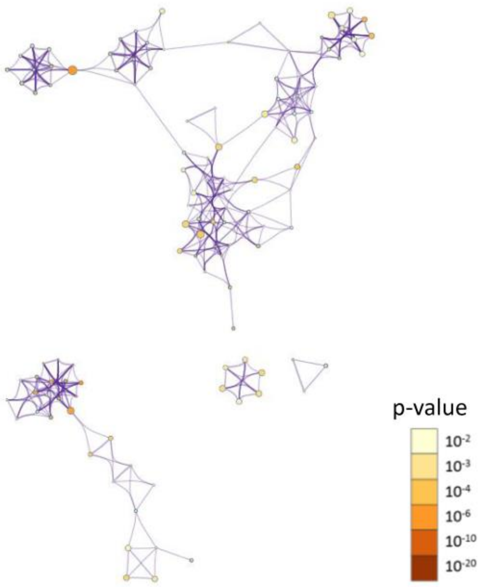

Figure S2: Specific clusters of endothelial cells based on single cell RNA-seq.

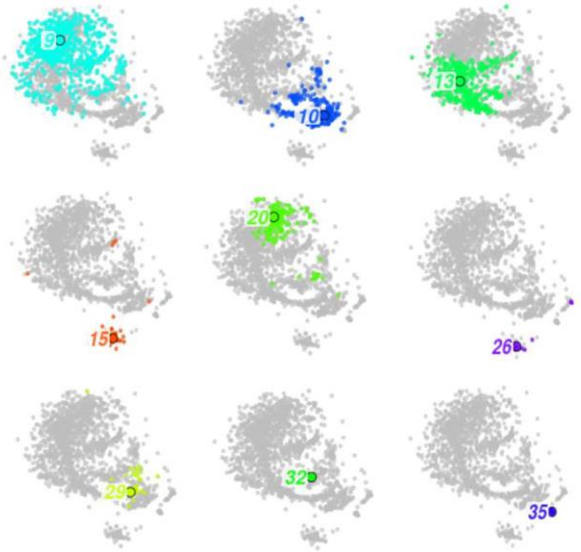

Table S1 Adjusted p value of the up- and down-regulated miRNAs.

ID	adj.P.Val	P.Value	t	B	logFC
hsa-miR-302c-3p	0.019	0.0000202	-11.02	2.81	-0.548781
hsa-miR-374b-3p	0.602	0.0018822	5.05	-2.32	0.015943
hsa-miR-501-5p	0.602	0.0019231	-5.03	-2.34	-0.012228
hsa-miR-140-5p	0.989	0.0047942	4.21	-3.36	0.058116
hsa-miR-181a-3p	0.989	0.0280933	2.82	-5.28	0.130995
hsa-miR-146a-5p	0.989	0.0310371	2.75	-5.39	0.229854
hsa-miR-369-5p	0.989	0.0365991	-2.63	-5.56	-0.282565
hsa-miR-671-3p	0.989	0.0383423	-2.59	-5.61	-0.005437
hsa-miR-887-3p	0.989	0.0409927	-2.55	-5.68	-0.028849
hsa-miR-29b-1-3p	0.989	0.0416934	2.54	-5.7	0.135783
hsa-miR-15a-5p	0.989	0.0421936	-2.53	-5.71	-0.011877
hsa-miR-302d-3p	0.989	0.0496785	-2.41	-5.88	-0.063768
